# Homology of the Dental Tissues

**Published:** 1877-08

**Authors:** 


### EDITORIAL.

### HOMOLOGY OF THE DENTAL TISSUES.

We published in the June number of the REGISTER from the *Dental Cosmos*, a part of Dr. A. H. Thompson's paper upon "*The Homology of the Dental Tissues etc.*," and in the present number the remaining part of the paper. The Doctor here persents with considerable degree of vigor an opinion, that has been entertained and somewhat agitated by a few in the Dental Pro fession.

This is a subject upon which much may be said upon both sides. It is one however that lies outside of the domain of demonstration. But we would not condemn the discussion of any thing occupying such a position.

While we do not concur in all the positions taken in the article, and perhaps hardly with the tenor of its leading thought, yet we have read the article with great interest, and would recommend its careful perusal to our readers; it affords matter for thought.

